# Spontaneous Resolution of Extensive Iatrogenic Type A Aortic Dissection After Transcatheter Aortic Valve Replacement

**DOI:** 10.1016/j.jaccas.2021.12.009

**Published:** 2022-04-20

**Authors:** Jino Park, Seung-Ah Lee, Do-Yoon Kang, Ho Jin Kim, Jung-Min Ahn, Joon Bum Kim, Duk-Woo Park, Suk Jung Choo, Seung-Jung Park, Dae-Hee Kim

**Affiliations:** a Department of Cardiology, Asan Medical Center, College of Medicine, University of Ulsan, Seoul, Republic of Korea; b Department of Thoracic and Cardiac Surgery, Asan Medical Center, College of Medicine, University of Ulsan, Seoul, Republic of Korea

**Keywords:** aortic dissection, aortic valve stenosis, transcatheter aortic valve replacement, AV, aortic valve, CT, computed tomography, EF, ejection fraction, POD, postoperative day, RCA, right coronary artery, TAAD, type A aortic dissection, TAVR, transcatheter aortic valve replacement, TEE, transesophageal echocardiography

## Abstract

The management of type A aortic dissection (TAAD) during transcatheter aortic valve replacement (TAVR) is challenging because TAVR is often performed in elderly patients with significant surgical risk. We present a case of extensive TAAD that developed during the TAVR procedure, which resolved spontaneously with medical treatment. (**Level of Difficulty: Intermediate**)

## Central Illustration

**Figure:**
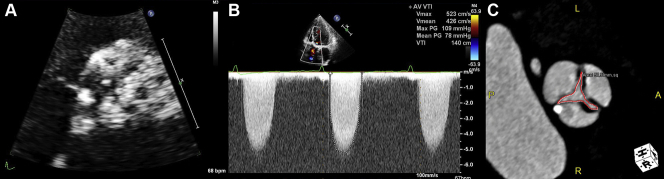

**Figure 1:**
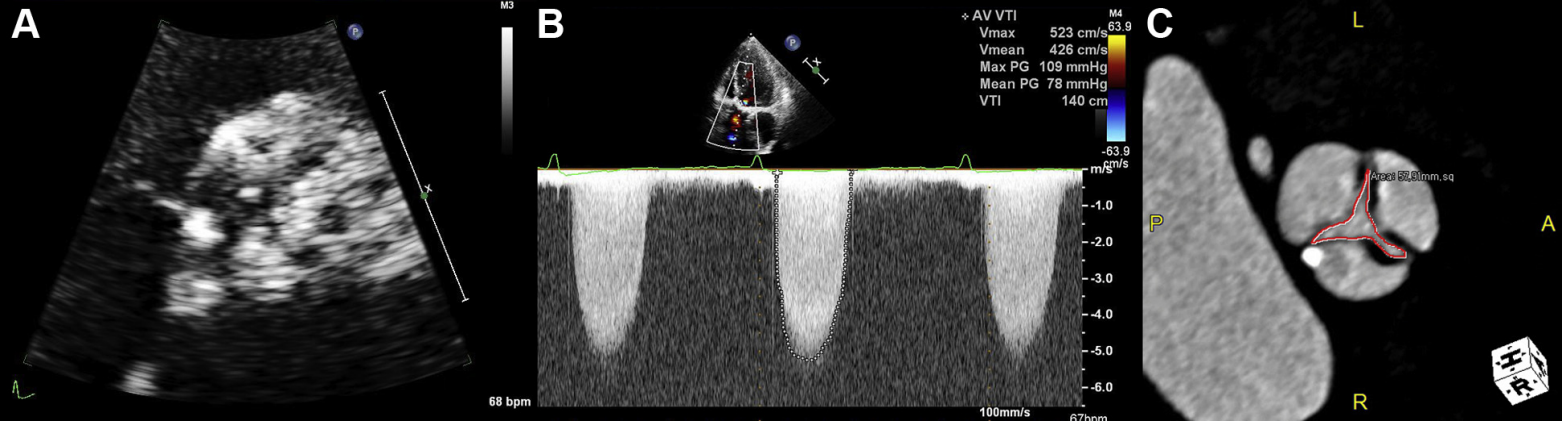
Baseline Transthoracic Echocardiography and Computed Tomography Images Transthoracic echocardiography images demonstrate aortic stenosis with severe degenerative change **(A)** and an aortic valve (AV) peak velocity of 5.2 m/s and mean pressure gradient of 78 mm Hg **(B)**. Computed tomography image shows AV area of 0.6 cm^2^ by planimetry **(C)**.

A 78-year-old woman presented with progressive exertional dyspnea. Her blood pressure and pulse rate, measured at the outpatient clinic, were 110/70 mm Hg and 70 beats/min, respectively. Grade 5 ejection systolic murmur was noted and was best heard in the aortic area. Transthoracic echocardiography (TTE) revealed severe degenerative aortic stenosis (aortic valve [AV] area 0.4 cm^2^, AV peak velocity 5.2 min/s) with moderate left ventricle dysfunction (ejection fraction [EF] 42%) (Figure 1). Coronary angiography did not reveal significant diseases.

She refused surgery, and our heart team decided to perform transcatheter aortic valve replacement (TAVR). The Society of Thoracic Surgeons Predicted Risk of Mortality was 4.5%. According to our center’s size selection criteria (an area-driven annulus diameter of 25.8 mm and an area of 522 mm^2^ measured by computed tomography [CT]), we selected a 26-mm Sapien XT (Edwards Lifesciences) valve.

TAVR was performed with the patent under general anesthesia via a femoral artery access, and the device was deployed with nominal volume. After deployment, transesophageal echocardiography (TEE) revealed residual mild paravalvular leakage (PVL). Additional post-dilatation was performed by overfilling a 23-mm balloon with 2 mL of contrast medium. PVL decreased slightly after dilatation. However, after 2 minutes, TEE revealed that the intimal flap originated from the aortic root.

## Medical History

The patient had a history of diabetes and was taking metformin, sitagliptin, and atorvastatin.

## Differential Diagnosis

Retrograde extension of type B dissection and type A aortic dissection (TAAD).

## Investigations

**Figure 2:**
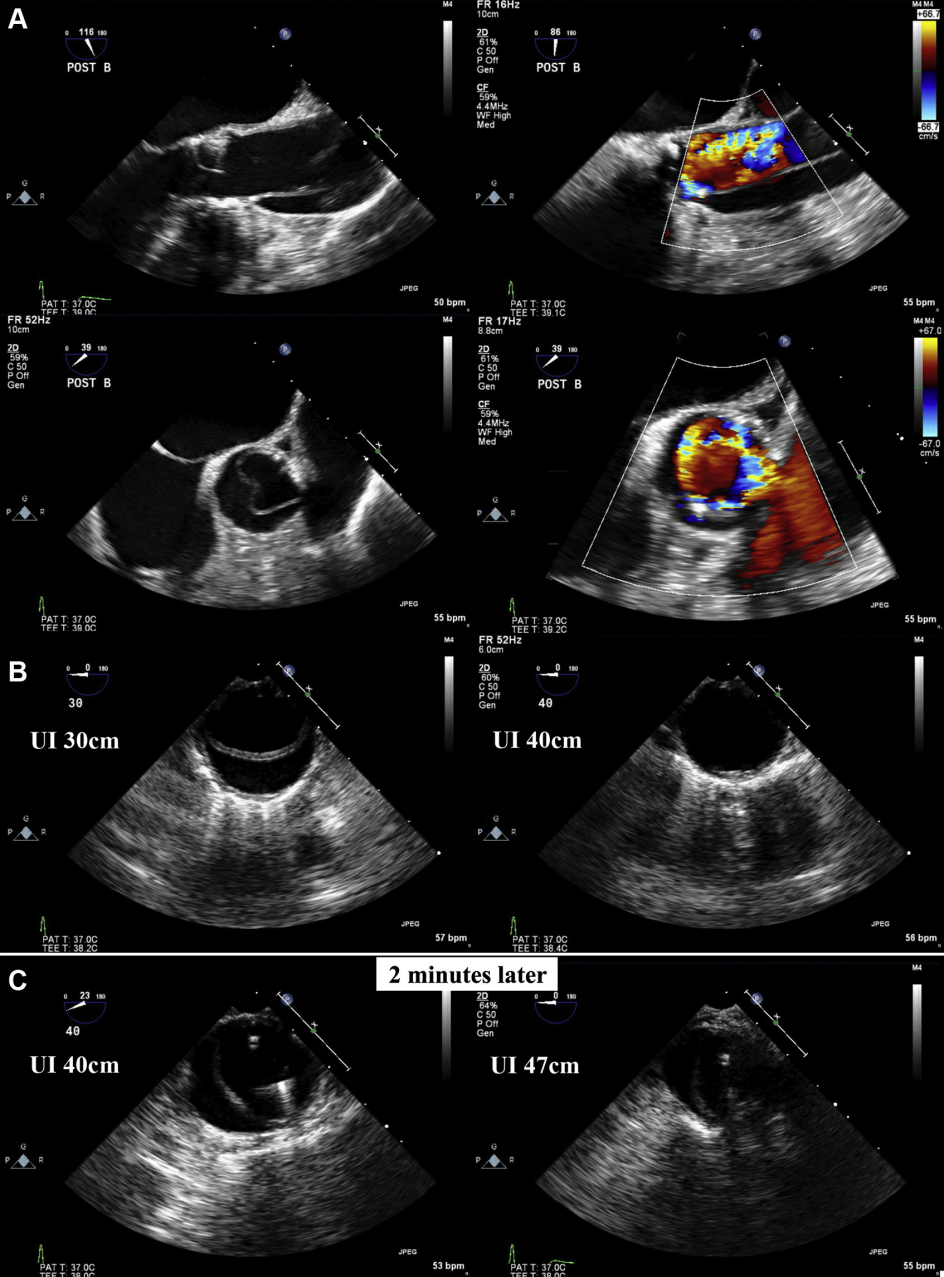
Transthoracic Echocardiography Images Immediately After Transthoracic Aortic Valve Replacement Transthoracic echocardiography images show the type A aortic dissection originating from the aortic root **(A)** and extending up to the descending thoracic aorta **(B)**. Further distal propagation of the type A aortic dissection up to the abdominal aorta can be observed **(C)**. UI = upper incisor.

TEE revealed the propagation of TAAD (Figure 2A, Video 1). The intimal flap originated from the aortic root near the right coronary artery (RCA) ostium and propagated to the descending thoracic aorta at 30 cm from the incisors (Figure 2B). After another 2 minutes, further progression of the TAAD was observed up to the abdominal aorta at 47 cm from the incisors (Figure 2C, Video 2). However, the patient was in hemodynamically stable condition, and pericardial effusion was absent.

## Management

After diagnosing TAAD, we considered open surgery after discussion with the heart team. However, the patient’s family refused, and conservative treatment was chosen. She was monitored in the cardiac care unit under strict blood pressure and heart rate controls with intravenous labetalol and nicardipine infusion. Dual antiplatelet therapy was discontinued. She was asymptomatic, with stable vital signs. Neurologic manifestations and end-organ perfusion abnormalities (monitored by serum creatinine, liver, and cardiac enzyme levels) were not observed.

**Figure 3:**
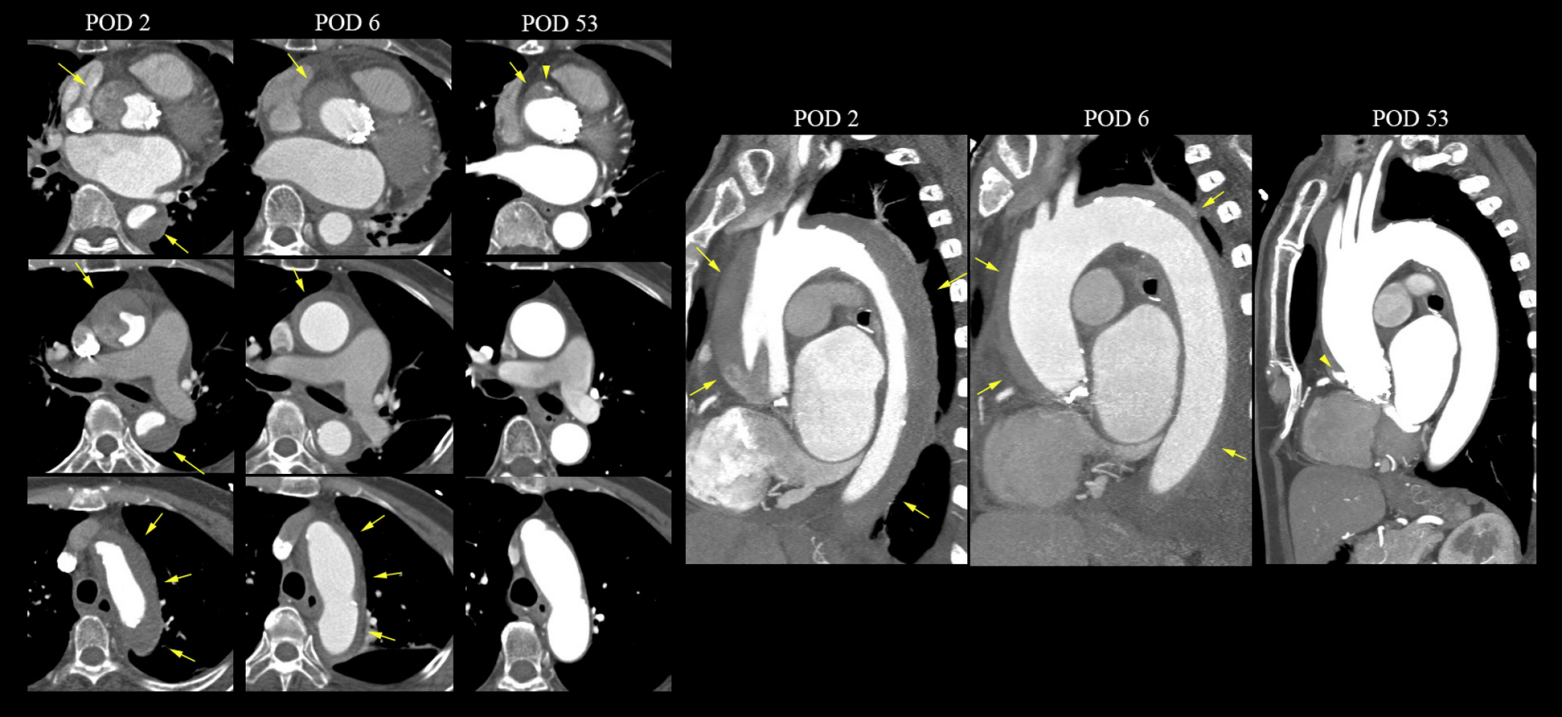
Serial Follow-Up Computed Tomography Images Serial follow-up computed tomography demonstrates the gradual resolution of the type A aortic dissection. Computed tomography on POD 2 reveals type A aortic dissection with the incompletely thrombosed false lumen **(arrows)** extending from the aortic root to the abdominal aorta. Computed tomography on postoperative day (POD) 53 shows the thrombosed false lumen resolution, leaving only a small focal outpouching contrast-filled lesion near the right coronary artery ostium **(arrowheads)**.

**Figure 4:**
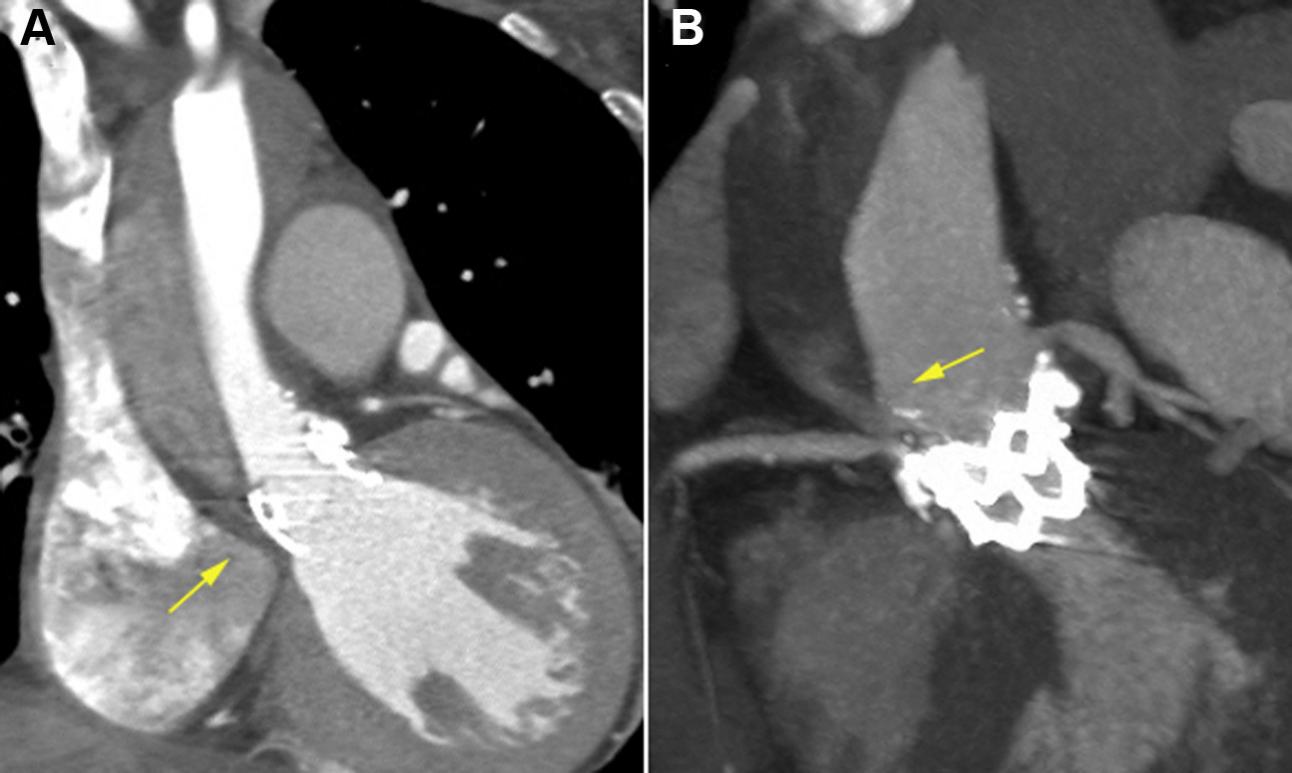
Computed Tomography Images Showing the Site of Intimal Tear Postoperative day 2 computed tomography images reveal the dissection flap originating from the aortic wall covered with the stent frame **(A)** and propagating spirally through the aortic wall above the right coronary artery ostium **(B)**.

Postoperative day (POD) 2 CT revealed TAAD that extended from the aortic root to the ostium of the superior mesenteric artery without the compromise of blood flow to the branch vessels, including the coronary and neck vessels (Figure 3, Video 3). The entry site of the dissection flap was located near the RCA ostium (Figure 4).

**Figure 5:**
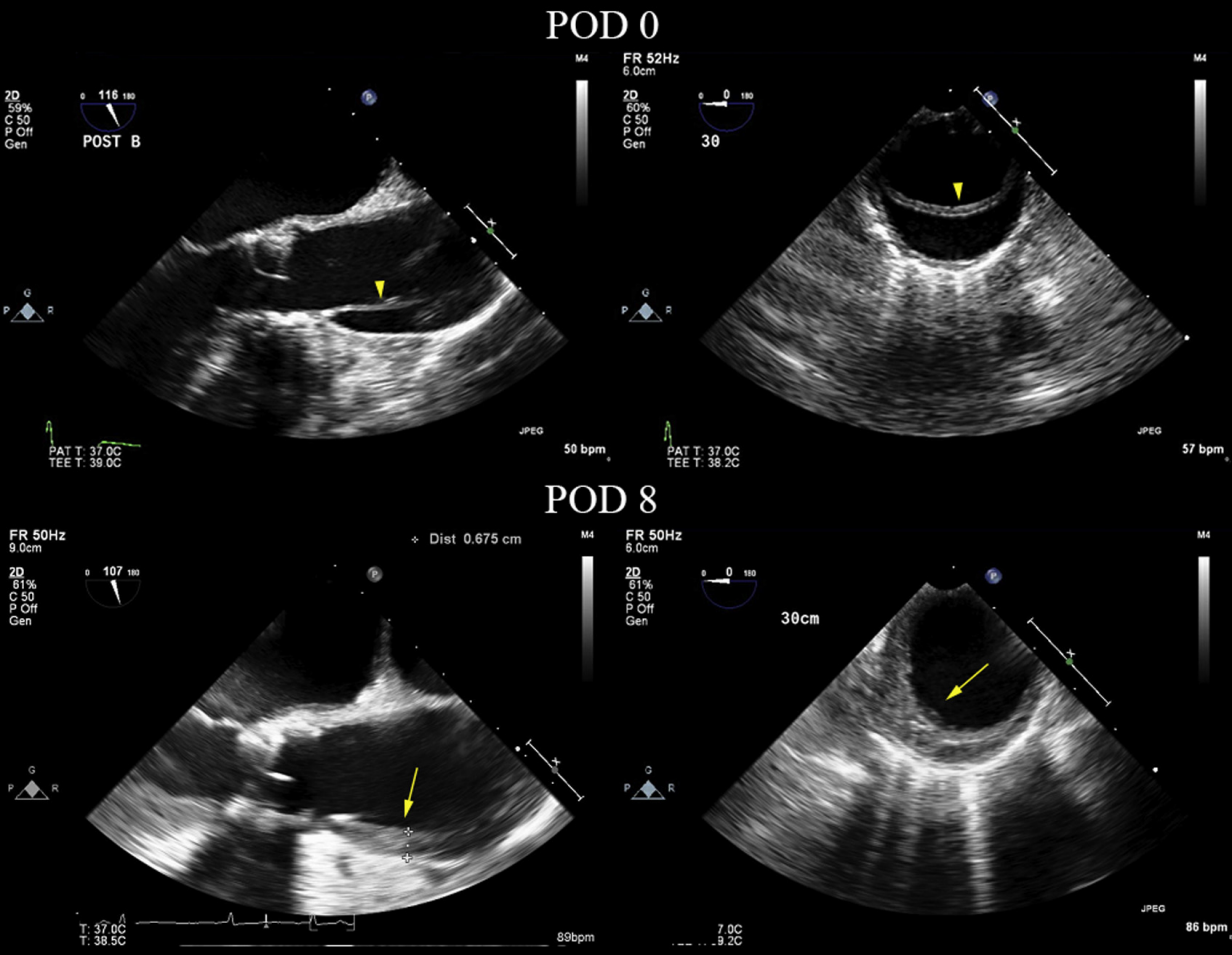
Serial Follow-Up of the Transesophageal Echocardiography Images Serial follow-up transesophageal echocardiography images show the intimal flap **(arrowheads)** originating from the aortic root after device implantation and a compactly thrombosed false lumen **(arrows)** on postoperative day (POD) 8.

Her postprocedural course remained uneventful, and she was transferred to the general ward. The aortic dissection (AD) was serially followed up by CT, TTE, and TEE. Fortunately, during the 3-week follow-up, these imaging evaluations demonstrated that the false lumen was thrombosed completely (Figures 3 and 5). The size of the thrombosed false lumen gradually decreased, suggesting TAAD resolution. TTE showed a well-functioning prosthetic valve with trivial PVL, and left ventricle systolic function was normalized. Eventually, she was discharged on POD 22.

## Discussion

AD is an infrequent (0.2%) complication of TAVR,^1^ and intimal disruption by guidewire or catheter manipulation, heavy calcification, suboptimal measurements of the AV complex, aggressive balloon valvuloplasty, an excessively oversized valve, and difficult valve positioning can be risk factors.^2^ In our case, AD developed after balloon dilatation for reducing the PVL. The delivery balloon was inflated to a diameter of 27 mm, which was approximately 7% oversized than the measured annular area. Approximately 5% to 15% of oversizing is optimal when the AV complex is not heavily calcified. However, the ratio between the sinotubular junction and the annular area was 1.14, which indicates a relatively small sinotubular junction size vulnerable to balloon injury.

Owing to the high early mortality of medically managed TAAD compared with surgical management, standard management classically includes open surgical repair.^2^ However, this is challenging in TAVR patients, who are generally older and have a high operative risk, which causes a treatment dilemma. To date, there are limited data and no definite guidelines on the management of AD during TAVR.

Upon literature review, we found several case reports of AD during TAVR. There were reports of surgically treated cases with good outcomes, whereas others resulted in mortality.^3^^,^^4^ Endovascular therapy had a remarkable outcome in several cases, but its application was limited to type B AD.^5^ Few patients had recovered with medical treatment; as in our case,^6^^,^^7^ they were in hemodynamically stable condition and had no pericardial effusion or branch vessel compromise. However, we did not find any cases presenting the natural course of extensive TAAD immediately after TAVR with complete serial imaging data, and the mechanism of spontaneous resolution remains unclear. The most plausible explanation is an interaction between the stent frame and intimal tear site near the RCA ostium; partial clogging and stabilization effects could be suggested (Figure 4).

In our case, the rapid progression of iatrogenic AD was detected by the intraprocedural TEE immediately after dilatation. Although the minimalist approach is currently mainstream, TEE should always be available on demand because it facilitates a prompt diagnosis and proper decision making.^2^

## Follow-Up

The patient visited the outpatient clinic 2 months after TAVR. She was asymptomatic and showed a better performance status. Follow-up CT and TTE revealed an almost-resolved, thrombosed false aortic lumen, with only a small focal outpouching contrast-filled lesion near the previous entry site (Figure 3, Video 4). Recently, we confirmed by a telephone interview that the patient was alive 6 years after TAVR and that she was doing well.

## Conclusions

TAAD during TAVR is a rare but catastrophic complication. Careful preprocedural evaluation of the AV complex with optimal sizing is essential for its prevention. Although surgical intervention is the standard treatment for TAAD, conservative treatment could be an acceptable option in selected cases. We recommend caution and discretion when generalizing the management of our case to other TAAD cases.

**Learning Objectives**

• To understand the importance of preprocedural evaluation of the AV complex and optimal sizing in TAVR.
• To understand the natural course of TAAD during TAVR using serial imaging and review feasible treatment options.
• To highlight the importance of intraprocedural TEE for the rapid diagnosis and management of TAVR complications.

## Funding Support and Author Disclosures

The authors have reported that they have no relationships relevant to the contents of this paper to disclose.

## Appendix

Video 1 A video clip of Figure 2A.

Video 2 A video clip of Figures 2B and 2C.

Video 3 Rearranged video clip from CT images at POD 2.

Video 4 Rearranged video clip from CT images at POD 53.

## References

[1] Walther T., Hamm C.W., Schuler G. (2015). Perioperative results and complications in 15,964 transcatheter aortic valve replacements: prospective data from the GARY registry. J Am Coll Cardiol.

[2] Langer N.B., Hamid N.B., Nazif T.M. (2017). Injuries to the aorta, aortic annulus, and left ventricle during transcatheter aortic valve replacement: management and outcomes. Circ Cardiovasc Interv.

[3] D’Onofrio A., Tessari C., Bianco R., Isabella G., Di Gregorio G., Gerosa G. (2012). Acute ascending aortic dissection during transaortic balloon-expandable aortic valve implantation. J Thorac Cardiovasc Surg.

[4] Pontious M.E., Ashfaq A., Watson J.J. (2020). Late type A dissection after transfemoral aortic valve replacement. J Am Coll Cardiol Case Rep.

[5] Baikoussis N.G., Argiriou M., Kratimenos T., Karameri V., Dedeilias P. (2016). Iatrogenic dissection of the descending aorta: conservative or endovascular treatment?. Ann Card Anaesth.

[6] Miha S., Rok Z., Nikola L., Matjaž B. (2018). Aortic dissection after transcatheter aortic valve replacement: conservative approach with good outcome. J Clin Case Rep.

[7] Zhu Q., Sondergaard L., Liu X., Wang J. (2021). Iatrogenic type-A aortic dissection due to transcatheter aortic valve implantation. Eur Heart J Case Rep.

